# Robust mosquito species identification from diverse body and wing images using deep learning

**DOI:** 10.1186/s13071-024-06459-3

**Published:** 2024-09-02

**Authors:** Kristopher Nolte, Felix Gregor Sauer, Jan Baumbach, Philip Kollmannsberger, Christian Lins, Renke Lühken

**Affiliations:** 1 Bernhard Nocht Institute for Tropical Medicine, Hamburg, Germany; 2 Institute for Computational Biology, University of Hamburg, Hamburg, Germany; 3 Biomedical Physics, Heinrich Heine University Düsseldorf, Düsseldorf, Germany; 4 Faculty of Engineering and Computer Science, Hamburg University of Applied Sciences, Hamburg, Germany

**Keywords:** Artificial intelligence, Entomology, Mosquitoes, Convolutional neural network

## Abstract

Mosquito-borne diseases are a major global health threat. Traditional morphological or molecular methods for identifying mosquito species often require specialized expertise or expensive laboratory equipment. The use of convolutional neural networks (CNNs) to identify mosquito species based on images may offer a promising alternative, but their practical implementation often remains limited. This study explores the applicability of CNNs in classifying mosquito species. It compares the efficacy of body and wing depictions across three image collection methods: a smartphone, macro-lens attached to a smartphone and a professional stereomicroscope. The study included 796 specimens of four morphologically similar *Aedes* species, *Aedes aegypti*, *Ae. albopictus*, *Ae. koreicus* and *Ae. japonicus japonicus*. The findings of this study indicate that CNN models demonstrate superior performance in wing-based classification 87.6% (95% CI: 84.2–91.0) compared to body-based classification 78.9% (95% CI: 77.7–80.0). Nevertheless, there are notable limitations of CNNs as they perform reliably across multiple devices only when trained specifically on those devices, resulting in an average decline of mean accuracy by 14%, even with extensive image augmentation. Additionally, we also estimate the required training data volume for effective classification, noting a reduced requirement for wing-based classification compared to body-based methods. Our study underscores the viability of both body and wing classification methods for mosquito species identification while emphasizing the need to address practical constraints in developing accessible classification systems.

**Graphical abstract:**

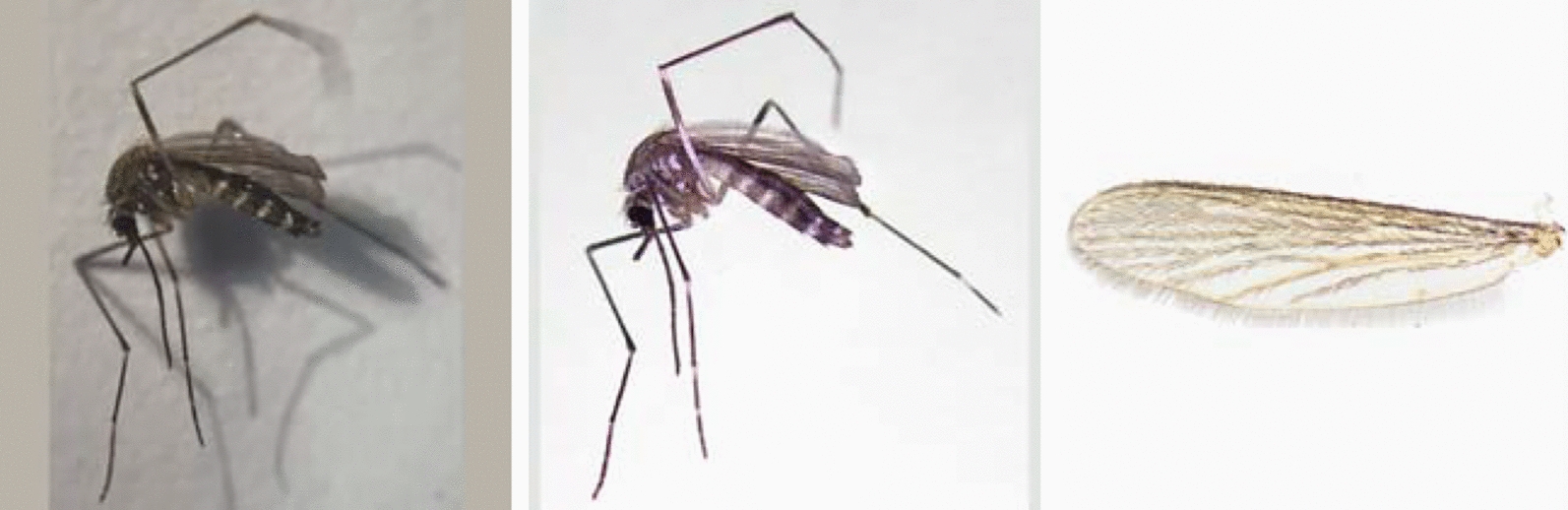

**Supplementary Information:**

The online version contains supplementary material available at 10.1186/s13071-024-06459-3.

## Background

Mosquito-borne diseases pose a significant global health risk, particularly in tropical and subtropical regions [1]. However, global change processes such as global warming and increased international trade have facilitated the spread of mosquitoes and their associated pathogens into previously unaffected regions. This emphasizes the need for effective vector surveillance programs [2]. Consequently, accurate species identification is crucial as mosquito species differ strongly in their medical and veterinary relevance. This is determined by species-specific differences in their vector capacity, e.g. ecology, behavior and vector competence. However, traditional morphological identification methods and molecular assays are costly and require specialized expertise [3].

Advancements in artificial intelligence, particularly convolutional neural networks (CNNs), offer potential to accurately identify mosquitoes based solely on images [4–8]. However, a significant gap remains between proof-of-concept studies and practical software applications for vector surveillance. Existing solutions are limited to the citizen science application MosquitoAlert, which still relies on manual confirmation and the commercial IDX imaging tower from the company VecTech [9, 10]. Moreover, the efficacy of models is often confined to controlled environments. For instance, most existing CNN models for mosquito species identification rely on a single imaging capture device [4–6, 11], potentially limiting their practical application because of sensitivity to variations in image conditions [12]. In addition, current CNN models primarily use images of the full mosquito body for classification, but limited attention has been given to wing images [7, 13]. The use of full body images is the straightforward approach as the preparation of wing images requires additional laboratory work. However, images of the nearly two-dimensional mosquito wings are easier to standardize, and from geometric morphometric studies it is well known that wing vein patterns are sufficient characteristics for the identification of mosquito species [14]. Yet, a direct comparison between images depicting the full body and wing images for mosquito species identification is missing.

To address these gaps in knowledge, we systematically compare the effectiveness of depictions of mosquito wings and full mosquito bodies for species identification. Additionally, we investigate the usability of different image capture systems, including smartphone, macro-lens attached to a smartphone and stereomicroscope.

## Methods

### Dataset construction

We collected images from 797 female mosquito specimens with 198–200 specimens of four different species: *Aedes aegypti*, *Ae. albopictus*, *Ae. koreicus* and *Ae. japonicus japonicus* (*Ae. japonicus*) (Table 1). All specimens were reared under standardized conditions in the arthropod rearing facility at the Bernhard Nocht Institute for Tropical Medicine, Hamburg. Each specimen was photographed using three different devices: a smartphone (iPhone SE 3rd Generation, Apple Inc., Cupertino, CA, USA), a macro-lens (Apexel-25MXH, Apexel, Shenzhen, China) connected to the same smartphone and a stereomicroscope (Olympus SZ61, Olympus, Tokyo, Japan) with an attached camera (Olympus DP23, Olympus, Tokyo, Japan). The images were captured in the TIF format and in 3024 × 3024 and 3088 × 2076 resolution for smartphone and stereomicroscope images, respectively. In the following text, we will refer to the smartphone as a “phone,” the smartphone with a macro-lens attachment as “macro-lens” or “macro” and the stereomicroscope as “microscope” or “micro.”

**Table 1:** Composition of the training, validation and testing datasets for both wing and body images

Depiction	Datasplit	Device	*Aedes aegypti*	*Aedes albopictus*	*Aedes japonicus*	*Aedes koreicus*	Total
Body	Testing	Phone	30	30	30	30	120
		Macro-lens	30	30	30	30	120
		Microscope	30	30	30	30	120
	Training	Phone	139	139	140	138	556
		Macro-lens	139	139	140	138	556
		Microscope	139	139	140	138	556
	Validation	Phone	30	30	30	30	120
		Macro-lens	30	30	30	30	120
		Microscope	30	30	30	30	120
	Total		597	597	600	594	2388
Wing	Testing	Macro-lens	30	30	30	30	120
		Microscope	30	30	30	30	120
	Training	Macro-lens	139	139	140	138	556
		Microscope	139	139	140	139	557
	Validation	Macro-lens	30	30	30	29	119
		Microscope	30	30	30	30	120
	Total		398	398	400	396	1592

For the “body” dataset, the complete mosquitoes were photographed with all three devices in the same orientation to guarantee the visibility of identical features in all the pictures (example images can be found in the Supplementary File 1). Subsequently, for the “wing” dataset, the left and right wings were mounted on a microscope slide using the embedding medium Euparal (Carl Roth, Karlsruhe, Germany) and photographed with the macro-lens and microscope. Due to the small size of the wings, image capture through the phone only was not feasible. The left wing of each specimen was used. If the left wing was damaged, the right wing was used as an alternative.

Image capture for *Ae. aegypti*, *Ae. albopictus* and *Ae. koreicus* involved capturing individual images in batches of 50 specimens before alternating to a different species to reduce biases during the image capture process, e.g. light conditions in the room. Images of *Ae. japonicus* were collected after the initial data collection process had been completed, because we aimed to add another morphologically similar species to the study to increase its robustness. All images were manually cropped to remove as much background as possible and subsequently downscaled to a size of 300 × 300 pixels. To create images with a ratio of 1:1, images were cropped with padding. The complete image dataset was randomly partitioned into training (70%), validation (15%) and testing (15%) subsets (Table 1). Thereby, the dataset split was determined based on mosquito specimen rather than individual images to ensure a stringent division between the datasets.

### Training pipeline

The CNN training pipeline was developed using the *Python* 3.10 and the libraries *Keras* and *TensorFlow* (both in version 2.14) [15, 16]. The training set was randomly augmented during training through pre-defined augmentation operations. Data augmentation artificially increases the size and diversity of the training set by applying reasonable image transformations. We utilized *RandomAugment* for color augmentation. Geometric augmentations were added through *RandomRotation, RandomTranslation* and *RandomFlip*. Additionally, we included augmentations informed by Geihros et al. [17] to reduce the texture bias and shift towards a shape bias as species identification through morphometric features is a task generally based on shapes and not textures. Therefore, we implemented *ColorDegeneration*, *RandomSharpness* and *GuassianNoise*. All augmentations were implemented through either the *Keras* or the *Tensorflow* libraries. Hyperparameters defining augmentation strength were not optimized by performance but selected before training based on visual cues so that important features in images can be recognized while still providing a high degree of variance.

EfficientNetV2B0 was selected as CNN architecture for its good performance on ImageNet and its comparatively fast training time [18, 19]. Two different learning strategies, transfer learning and fine tuning, were utilized to ease the training process and capitalize on pre-existing knowledge from pre-trained CNNs. We chose to investigate both methods because of their unique strengths and weaknesses. While fine-tuning generally enhances performance, it also increases the risk of overfitting, particularly when dealing with limited training data [20]. In transfer learning, a CNN model pre-trained on the ImageNet dataset served as a feature extractor, excluding the original classification head. Additional layers, including GlobalAveragePooling, Dropout and a Dense Layer, were added for classification. During transfer learning, the base model's weights were frozen, and only the newly introduced classification layers were trained on each dataset. Subsequently, fine-tuning was employed to further optimize the model's weights by unfreezing 50% of the feature extraction segment of the model.

### Depiction comparison

All images captured by macro-lens and microscope were used to compare the efficacy of CNN models trained on body and wing images. Phone images were excluded from this experiment, as they were only collected for the full mosquito body and not for the mosquito wings, which would prevent a direct comparison. We utilized the previously described pipeline by first transfer learning for 24 epochs and subsequent fine-tuning to 64 epochs. Early stopping was used to stop the training after 12 epochs without a decrease of validation loss. A complete list of the hyperparameters used to train the final models can be found in the supplement (refer to Supplementary File 1). A total of four models per depiction were trained with the same hyperparameters only differing between runs by the seed (3, 7, 9, 1), which defines the random aspects of the data pipeline, i.e. data shuffling and random augmentation. The mean accuracy of the four models per depiction is reported with 95% confidence interval (95% CI) on the testing set, consisting of macro- and microscope images of the full mosquito body and the mosquito wings, respectively.

### Data demand experiment

To determine the minimum amount of training data needed for reliable species classification by a CNN, models were trained with increasing quantities of images per depiction (body or wing), which were randomly sampled from the same dataset used in the depiction experiment. For each quantity (10, 20, 40, 80, 120, 160, 200, 240), we trained four models, each differing only in the seed (3, 7, 9, 1) used for data loading, shuffling and augmentation. The models underwent transfer learning for 24 epochs before fine-tuning 50% of the model for an additional 24 epochs. Hyperparameters and augmentation strategies were set as defined in the depiction experiment (refer to Supplementary File 1). The average accuracy per training quantity and its 95% CI on the testing set were reported.

### Device comparison

To investigate the impact of different image capture devices on model performance, the dataset was divided according to the device used for image capture. Subsequently, we trained four models on each depiction and device, with each model varying in the seed (3, 7, 9, 1) used for random data shuffling and augmentation. Models for classifying body images were trained on subsets captured by phone, macro-lens and microscope, while models classifying wing images were trained solely on subsets captured by macro-lens and microscope, as phone images were unavailable. The hyperparameters and augmentation strategies were defined according to the specifications outlined in the previous section (refer to Supplementary File 1). Given the limited training data, only transfer learning was employed. The mean accuracy and its 95% CI for the models trained on each depiction and device using the complete testing dataset are reported.

## Results

### Depiction comparison

**Fig. 1:**
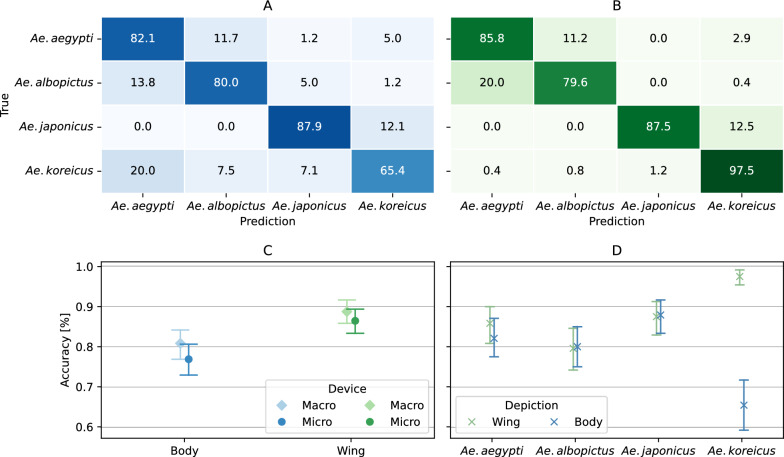
Average normalized confusion matrices illustrating the classification performance of body (**A**) and wing depictions (**B**). **C** Average accuracy with 95% CI for model performance on the testing set, categorized by image capture device. **D** Average accuracy with 95% CI for model performance on the testing set, categorized by species

Models trained and tested on body images achieved an average accuracy of 78.9% (95% CI: 77.7–80.0), while models trained and tested on wing images demonstrated a superior accuracy of 87.6% (95% CI: 84.2–91.0). All models trained on wings displayed better performance than models trained on bodies. The difference in performance varied between the different species. For *Ae. aegypti, Ae. albopictus* and *Ae. japonicus*, the difference in average accuracy was < 5% comparing wing to body model performance, while for *Ae. koreicus*, the difference in average accuracy (32.1%) was more pronounced and contributed heavily to worse overall performance of the body models (Fig. 1). The wing models mostly showed confusion between the relatively closely related pairs of *Ae. aegypti-Ae. albopictus* and *Ae. japonicas-Ae. koreicus*; the body model mostly confused *Ae. koreicus* with all the other species. Comparing the performance of the models on the different devices, there appears to be no stark difference in performance.

### Data demand experiment

In the data demand experiment, models trained by fine-tuning generally outperformed their transfer-learned counterpart.

Furthermore, models which were trained on wing images generally outperformed models trained on body images. Average model performance tends to increase with growing training data size. At up to 80 images per class, we observed a steep increase in performance; thereafter, the increase in performance per further included images was only modest. The performance gap between the two training methods was most pronounced at approximately 40 images per class, where transfer learning and fine-tuning resulted in a 11.9% and 15.5% improvement in wing classification performance, respectively. With increasing training size, the gap between wing and body classification shrank, but at no point did the body classification result in a better classification performance.

### Device comparison

To further investigate whether the image capture method affected the performance of the models, we trained the models on images captured by a single method.

**Fig. 2:**
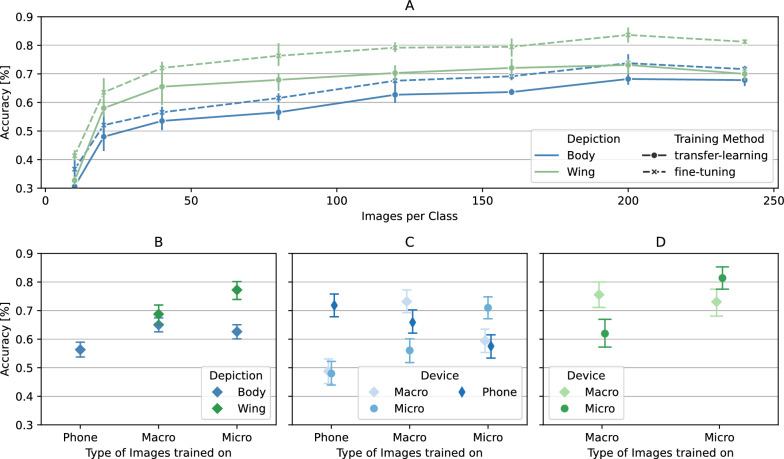
**A** Results from the data demand experiment. Average accuracy with 95% confidence intervals is given for both body and wing classification. Performance on the testing set for both transfer learning and fine-tuning is shown. Results from the device comparison experiment are presented as average accuracy with 95% CI. **B** Performance of models trained on phone, macro and micro, grouped by device. **C** Performance of body classification models trained on singular devices, categorized by image capture device in the testing set. **D** Performance of wing classification models trained on singular devices, categorized by image capture device in the testing set

The best performing models were the wing models trained on microcopy images, which achieved an average accuracy of 77.2% (95% CI: 70.3–84.1). The worst performing models were the body models trained on phone images, demonstrating an average accuracy 56.3% (95% CI: 51.1–61.6). When inspecting the models trained solely on one depiction, i.e. body or wing and different devices, the performance decreases on images captured with a device not included in the training data. The only exception from this were the body models trained solely on the images captured through a macro-lens, which also demonstrated good performance on images captured with a phone (Fig. 2).

## Discussion

The objective of this study was to compare the usefulness of different mosquito depictions (full body and wings) and image collection methods (smartphone, macro-lens attached to a smartphone and stereomicroscope) for mosquito species classification through CNNs. Furthermore, we estimated the minimum amount of training data needed to allow the models to reliably classify mosquito species.

In the depiction comparison experiment, the CNN models trained on wing images outperformed those trained on body images, exhibiting higher average accuracy. This was predominantly caused by the relatively low performance of the body models in classifying *Ae. koreicus*. The average wing classification accuracy was comparable to our previous work, which differentiated seven *Aedes* species and achieved a macro F1 score of 91% [13]. Body classification accuracy was significantly lower than reported in the literature [4–6, 8]. However, a direct comparison of the accuracy between the different CNN studies must be conducted with caution. The training sample size used in this experiment was considerably lower than that used in previous studies, and we did not employ optimization methods as increasing performance was not the main goal of this study. Instead, we wanted to systematically compare the effects of different mosquito depictions and image collection methods. Moreover, we compared four morphologically relatively similar and difficult to distinguish mosquito species, particularly *Ae. koreicus* and *Ae. japonicus* [21, 22], while other CNN studies focused on the classification of taxonomically distant mosquito species, which are also easier to distinguish by morphology [4, 8, 11].

We assume that wing images are superior to body images for training CNNs because of the reduced variance between images due to their nearly two-dimensional nature, providing more useful features for CNNs to extract for classification. Whole body classification introduces additional challenges, such as pose variations and complexities in color representation. As a result, we also observed that wing models required fewer training images to achieve comparable performance to the body classification system. The model performance starts to stagnate after approximately 80 images per class, in line with previous research [4]. However, it should be noted that the images in the data demand experiment were somewhat redundant including both microscope and macro-lens images.

Although wing preparation is a more laborious data collection process, the advantage of a lower data demand becomes particularly significant when integrating rare mosquito species. From ecological field studies, it is well known that the detection of rare species correlates with sampling effort, which is also evident in mosquito monitoring programs where a few species dominate while others are less abundant [23–26]. Therefore, reduced data requirements associated with wing images would allow the development of a reliable classification system capable of accurately identifying a wider range of mosquito species than body classification.

In the device experiment, a distinct correlation was observed between the devices used in the training data and the models' performance when tested on images with different devices. The trend persisted across all models in the experiment, suggesting that while CNN excelled with images from the same device as in the training data, their performance suffered with images from other devices. The inability of CNN to generalize across different settings, despite extensive augmentation, is a known weakness of the models and poses a significant challenge to their practical applicability in classification systems [12, 27, 28].

From our observations, two potential approaches for image capture devices emerge for the development of a mosquito species classification system. One possibility is a system that relies on strict standardization for both image collection and classification. Despite our attempts to standardize images, as seen with the slightly altered images of *Ae. japonicus*, achieving consistency even with a single device poses challenges. Therefore, the use of a predefined image capture device, such as a photo box, represents the most feasible approach for achieving image standardization [9]. Yet, this method incurs relatively high costs for image collection and could limit the accessibility of the system, particularly in resource-limited settings. In addition, the method is restricted to one device, making the method more difficult to establish under different settings. Alternatively, a classification system can be developed using a heterogeneous dataset with images captured by different devices. This approach enables the implementation of a generic identification model across diverse settings in vector research and surveillance without the need for specialized equipment. However, leveraging a heterogeneous dataset carries the risk of introducing biases into the model, such as device-related image characteristics, which may become discriminative features for classification [27]. Conversely, image preprocessing methods, such as removing background and lighting effects, could be applied to further standardize the images. In this regard, wing classification emerges as an ideal candidate because of the relatively simple shape of wings coupled with wing vein patterns serving as distinctly identifiable features.

In conclusion, the results of this study demonstrate that both wing and body images are suitable for CNN-based species classification even for closely related species. Thereby, the wings required fewer images than the bodies to yield reliable classification results. However, the use of different imaging devices can affect the CNN performance, which should be considered in future research to improve the practical applicability of the device.

### Supplementary Information

Supplementary file 1.

## Data Availability

Image data used for the training of the models were uploaded to DataDryad (https://doi.org/10.5061/dryad.b8gtht7mx).
