# Supplementary material for: Robust mosquito species identification from diverse body and wing images using deep learning
Source: Parasit Vectors. 2024 Sep 2;17:372. doi: 10.1186/s13071-024-06459-3 (PMC11370291; doi:10.1186/s13071-024-06459-3)
Supplement: Supplementary file 1 [file 13071_2024_6459_MOESM1_ESM.docx]

# **Appendix**

## **Image comparison**


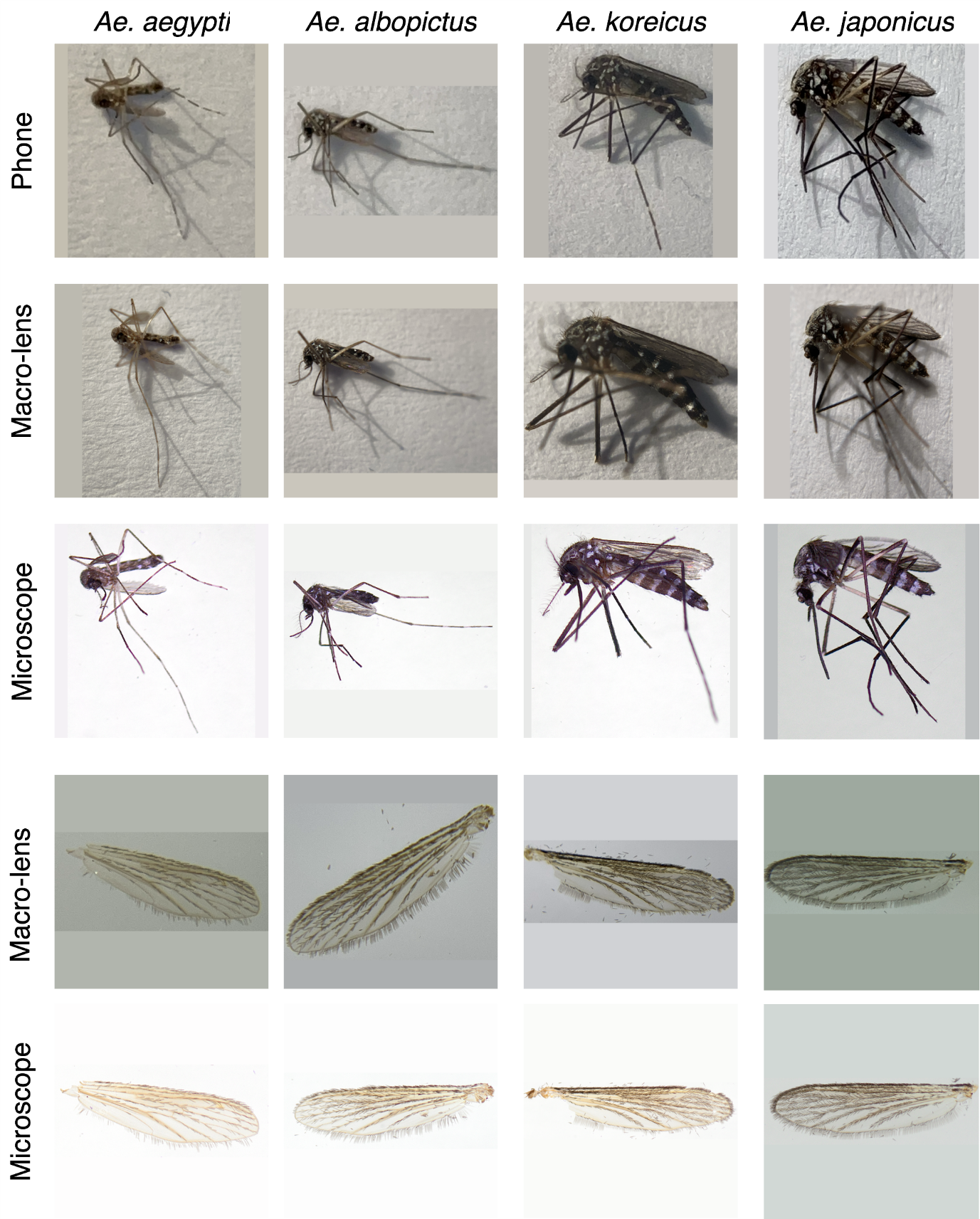


Appendix 1: Image of the samples with the identifier b50.

## **Hyperparameters**

Appendix 2: Hyperparameters used for the depiction, device and demand experiment for both body and wing classification.

| **Parameters** | **CNN** |
| --- | --- |
| Model Architecture | EfficientNetV2B0 |
| Image Resolution | 300x300 |
| Batch Size | 16 |
| Learning Rate Transfer | 0.0005 |
| Learning Rate FineTuning | 0.00001 |
| Early Stop Patience | 12 |
| RandAug Layers | 3 |
| RandAug Magnitude | 0.05 |
| RandomRotation Factor | 0.05 |
| RandomTranslation Factor | 0.05 |
| RandomColorDegeneration Factor | 0.25 |
| RandomSharpness Factor | 0.25 |
| GaussianNoise Factor | 0.1 |
| Label Smoothing | 0.1 |
| Dropout Probability | 0.5 |

## **GradCam**


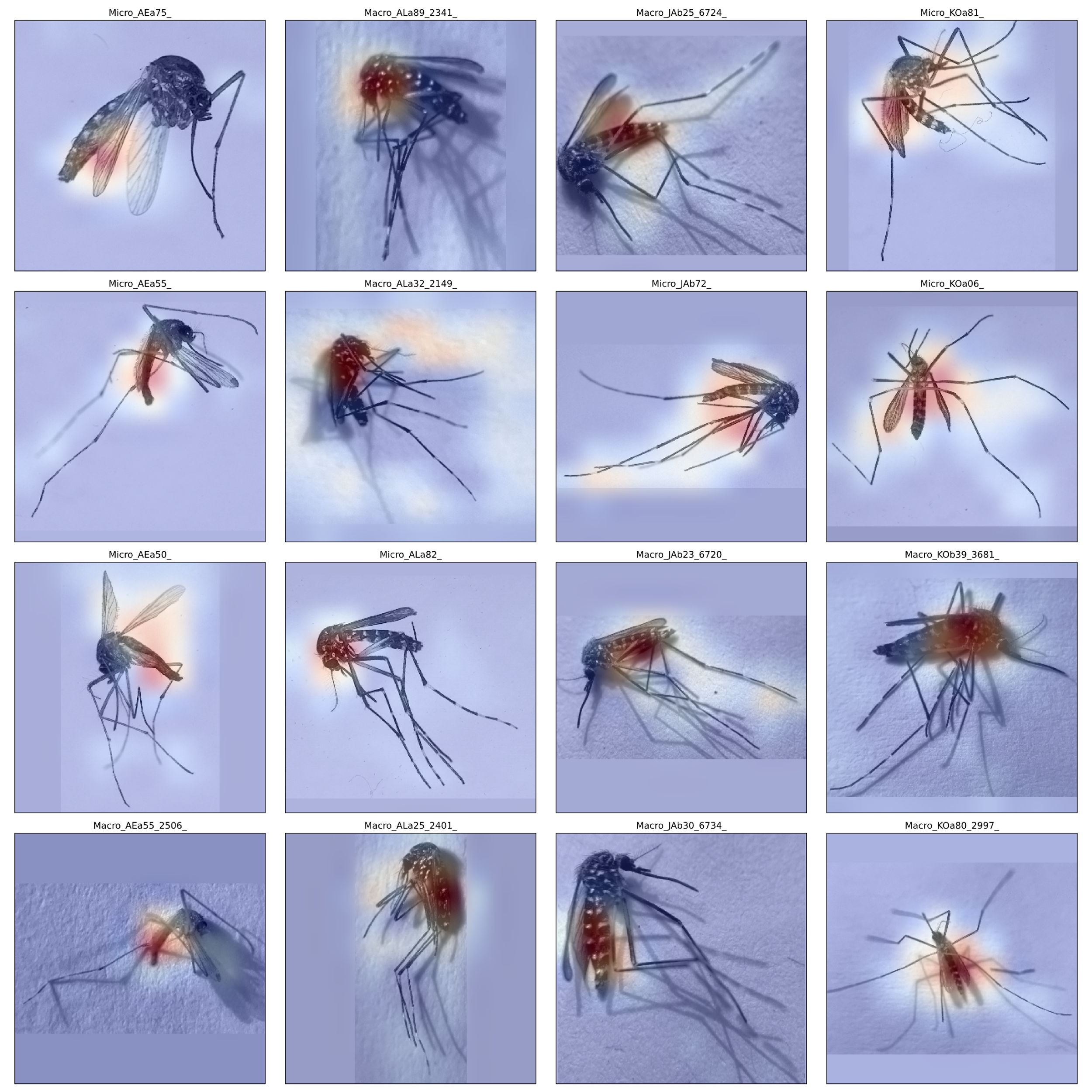


Appendix 3: Gradient weighted Class Activation Mapping (GradCam) generated from the body classifying model with the seed 9. All images are from successful prediction on the testing set. Red areas are the ones with the highest activation while blue areas are regions with the lowest relevance for classification. It can be observed that the model focuses on the thorax region for classification.


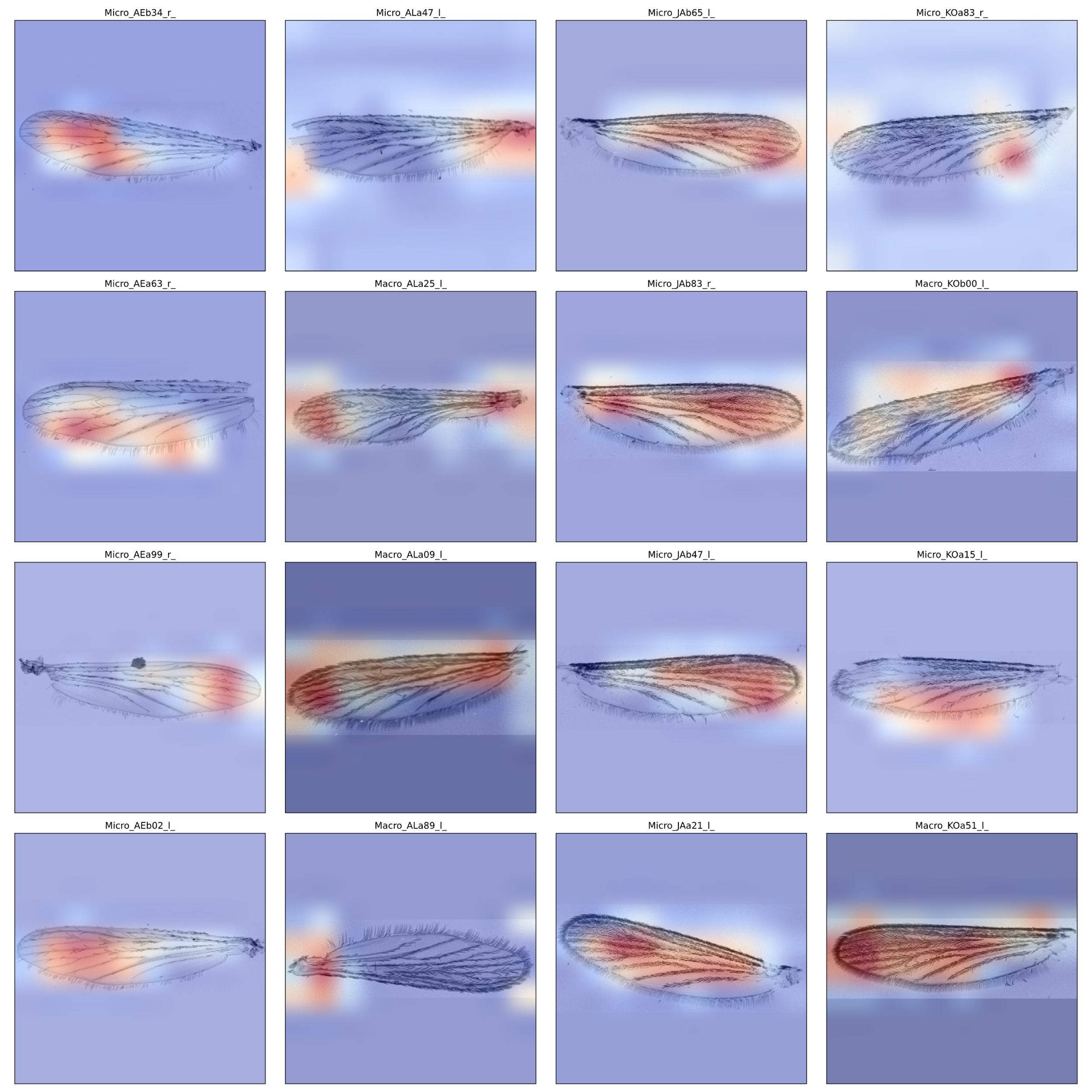


Appendix 4: Gradient weighted Class Activation Mapping (GradCam) generated from the wing classifying model with the seed 9. All images are from successful prediction on the testing set. Red areas are the ones with the highest activation while blue areas are regions with the lowest relevance for classification. It can be observed that the model often utilizes the whole wing for classification.
